# The spermatozoon neck role in infertility and intracytoplasmic sperm injection outcomes

**DOI:** 10.1007/s10815-025-03769-y

**Published:** 2026-01-05

**Authors:** Derek F. Kluczynski, Isabel Nester, Haley Prine, Kadyn Heising, Ethan Gartee, David Adegoke, Gunnar Eriksen, Conner Liber, Yashwanth Byreddy, Rudraksh Dua, Mira Adkins, Nathan Pan, Brady Artz, Natalie Doumet, Haley Salazar, Jakya Warren, Tomer Avidor-Reiss

**Affiliations:** 1 Department of Molecular, Cellular, and Developmental Biology, University of Toledo, Toledo, USA; 2 Department of Urology, University of Toledo Medical Center, Toledo, USA

**Keywords:** Acephalic spermatozoa, Intracytoplasmic sperm injection, Centriole, Spermatozoa neck, Infertility

## Abstract

The mammalian spermatozoon neck is a unique structure that functions during spermatid differentiation and spermatozoa swimming, and its contents are critical for post-fertilization embryogenesis. Mutations in proteins localizing to the neck connecting piece (the modified pericentriolar material) result in acephalic spermatozoa. In contrast, mutations in proteins localizing to the centriole often produce abnormal tail morphology. Acephalic spermatozoa can be categorized based on the exact location of the neck breakpoint. Here, we classify 24 proteins known to cause acephaly in human and mice spermatozoa into five different acephalic types, depending on where the breakpoint occurs. We also discuss other proteins found in the spermatozoon neck, which may result in spermatozoa acephaly. The relationship between the exact location of the neck’s break and intracytoplasmic sperm injection (ICSI) outcomes is explored in the context of the spermatozoon centrosome’s role. We conclude that to understand this relationship, future research should investigate DNA, phospholipase C zeta, and centriole functionality, in addition to the location of the acephalic breakpoint in the patient's sperm.

## Introduction

**Fig. 1:**
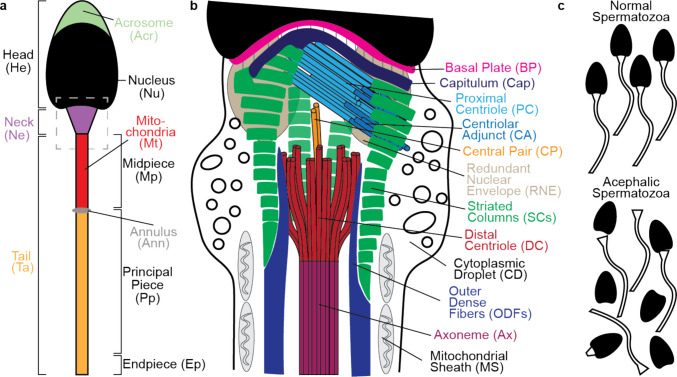
The human spermatozoon neck connects the head to the tail. (**a**) The spermatozoon is made up of the head, neck, and tail, with multiple substructures in each. (**b**) An ultrastructural representation of the spermatozoon neck, which includes multiple subunits. The head–tail connection is mediated via the basal plate (BP), capitulum (Cap), striated columns (SCs), and the two centrioles (PC and DC). (**c**) Representation of normal spermatozoa and acephalic spermatozoa

Spermatozoa leave the male reproductive tract to swim and reach an egg in the female reproductive tract, fertilizing it by depositing the essential ingredients that activates the egg and initiates embryogenesis [1]. To achieve this, spermatozoa are streamlined and consist of three functional units: the head, the neck (sometimes included within the midpiece), and the tail (Fig. 1a) [2].

The head contains at least two critical paternal factors: DNA [3] and phospholipase C zeta (PLCζ) [4], which are essential for fertilization. Intact DNA is vital for the embryo's genetic code, and its fragmentation can cause abnormal embryonic development [5–7]. It has been suggested that primarily double-stranded DNA breaks can cause fertilization/pregnancy failure by delaying the first embryonic division and morula stage development [8, 9]. Sperm DNA fragmentation can be examined using various methods, such as terminal deoxynucleotidyl transferase dUTP nick end labeling (TUNEL), single cell gel electrophoresis (aka Comet), Sperm Chromatin Dispersion (SCD), and Sperm Chromatin Structure Assay (SCSA) assays, which may have clinical significance, as demonstrated in three of four case reports where interventions led to a decrease in sperm DNA fragmentation [7]. PLCζ is essential for fertilization by inducing the calcium oscillations that are essential for egg activation and its dysfunction results in fertilization failure [10, 11].

The neck is a region at the junction of the head and tail, connecting them through unique structures found only in spermatozoa; it is also known as the head–tail coupling apparatus (HTCA) [12]. While clinically, the neck may be included in the midpiece, it is structurally and functionally distinct from the midpiece; therefore, for this review, we will refer to the junction between the head and tail as the neck [13, 14]. The neck is thought to regulate spermatozoa motility via the basal sliding hypothesis [15], which postulates that the force of axonemal microtubule doublet sliding is transmitted to the head via the connecting piece (neck). More recently, the neck has been proposed to function as a transmission system that mediates head kinking through the dynamic basal complex hypothesis [16], which explains that the distal centriole and surrounding modified pericentriolar material link tail beating to head kinking. Finally, the neck structures are essential in the zygote for forming the centrosome and originating embryonic centrioles [17, 18]. While significant progress has been made in understanding the structure, function, and composition of the neck, the precise mechanisms underlying these remain to be discovered.

Several recent reviews have discussed various aspects of the spermatozoon neck, including its protein composition [19], its centrioles [13, 20, 21], the transition of canonical centrioles to a specialized spermatozoon connecting piece [12], and its role in acephalic spermatozoa [22, 23]. This review will focus on the neck and its substructures, as well as the surrounding structures that are essential for normal spermatozoa morphology. We discuss the protein composition of the spermatozoon neck and the phenotypes observed when neck proteins are mutated, primarily in humans and mice, as they are the most prevalent in genetic and mutant studies. The purpose of this review is to provide intracytoplasmic sperm injection (ICSI) operators with a deeper understanding of the complexities associated with acephalic spermatozoa and to highlight the need for more diagnostic testing to improve prognosis for patients. We test the hypothesis that the spermatozoon head must be attached to both a functional proximal centriole and a functional distal centriole, and that other spermatozoon contributions, such as DNA and phospholipase C zeta, must be normal for ICSI to produce an offspring.

## Main Text

### Multiple cytoskeleton structures connect the head to the tail

The mammalian spermatozoon neck is a complex region comprising multiple structures revealed from the dawn of electron microscopy (Fig. 1b) [24–28]. Rostrally (toward the head), the neck borders the nucleus, which makes up most of the head. Caudally (toward the tail), the neck borders the tail midpiece, which contains the axoneme (Ax), the outer dense fibers (ODFs), and the mitochondrial sheath (MS). The neck is made of two main components. One, the poorly understood redundant nuclear envelope (RNE), which is outside the current scope of this review, as it does not appear to have a structural role in connecting the head to the tail [29–31]. Second, the spermatozoon centrosome and associated structures, such as the basal plate (BP) and outer dense fibers, which are essential for the head–tail connection (Fig. 1b) [13]. Additionally, while not always localized to the neck, many spermatozoa contain a cytoplasmic droplet (CD), a dilated cytoplasmic area viewed as residual cytoplasm that contains numerous sac-like structures [32, 33].

Below, we describe the main structures connecting the head to the tail in human spermatozoa, including the basal plate, various substructures of the centrosome, and briefly mention the head and tail structures to which they are attached: the nuclear membrane, the axoneme, and the outer dense fibers.

The head is connected caudally via the nuclear membrane to the basal plate, a structure that appears as an electron-dense plate in electron microscopy work just below the nuclear membrane [34]. SUN5 is a nuclear membrane protein that may extend into the basal plate [35, 36], and two proteins are implicated in being basal plate proteins: Nesprin3 [35] and BAG5 [37].

The basal plate, found just caudally to the nuclear membrane, connects caudally to the centrosome’s capitulum (Cap) via thin-fiber bridges (Fig. 1b) [26, 38]. The bridge’s composition is unknown. Still, they may include SUN-domain proteins (Sad1p and UNC-84 homologs) and outer nuclear membrane proteins of the KASH-domain family (Klarsicht, ANC-1, and Syne homologs) [12].

The spermatozoon centrosome comprises four primary substructures: the capitulum and striated columns (SCs), which are collectively referred to as the connecting piece, the proximal centriole (PC), and the distal centriole (DC) (Fig. 1b). The capitulum has also been referred to as the capitellum or capitella in the literature. The nature of the capitulum is unclear. Some describe it as a transient structure that disappears in mature spermatozoa of humans, mice, and rabbits [27]. In other mammals, the capitulum may be better described as the material surrounding the proximal centriole [26]. Earlier research also claims that the capitulum is a result of the striated columns fusing together [39]. The striated columns are composed of nine elongated, dense fibers that appear striated, with dark and pale regions visible from a side view, and small bridges that connect the dark areas [26]. The striated columns have also been referred to as segmented columns and a striated collar in the literature. The capitulum and striated columns are best known in mammals. Still, electron microscopy studies have found related structures (capitulum-like, striated column-like structures, and non-striated column-like structures) around the centrioles in the spermatids or spermatozoa of reptiles [40, 41] and birds [42, 43].

The capitulum and striated columns are thought to be derived from the centrioles and pericentriolar material surrounding them during spermiogenesis, based on electron microscopy (Fig. 1b) [26]. These two structures contain many centrosomal proteins, as determined by immunofluorescent studies [44]. Structurally, the connecting piece forms the walls of two “vaults,” one of which houses the typical proximal centriole, and the other houses the atypical distal centriole [27, 45]. Therefore, we refer to the capitulum and striated column as modified pericentriolar material based on electron microscopy and immunofluorescent studies.

The proximal centriole of the spermatozoon is an evolutionarily conserved structure that resembles the canonical centrioles of somatic cells. It is composed of nine sets of microtubule triplets [46]. It is thought that the proximal centriole is the mother centriole in the spermatozoon neck [47]. The proximal centriole also contains a remnant cilium called the centriolar adjunct (CA), made up of single and doublet microtubules [48]. The centriolar adjunct may influence fertility, with a longer centriolar adjunct potentially being detrimental to fertility outcomes [49].

The distal centriole (aka the flagellar basal body) is a reorganized fan-shaped centriole that is modified during spermiogenesis [44]. It contains nine sets of microtubule doublets with centriolar luminal proteins that are reorganized into rods. The distal centriole exhibits left–right asymmetry [16, 46] and connects to the spermatozoon’s axoneme, with the microtubule central pair (CP) running up through the distal centriole (Fig. 1b). The distal centriole is also considered the primary driver of the dynamic basal complex, with the left and right sides moving in opposition to each other. Interestingly, while many researchers have thought that the distal centriole degraded in mature spermatozoa, Zamboni & Stefanini [27] hinted towards the distal centriole being present in the mature spermatozoon, in 1971, but undergoing “profound modifications” before Fishman and colleagues’ discovery in 2018 [44].

The capitulum and striated columns are connected and share protein composition. The capitulum connects to the striated columns via an electrodense material (Fig. 1b) [26]. Both structures share proteins ODF1 and ODF2 [12, 50]. The capitulum forms the rostral ceiling, and the striated columns form the side walls. The striated columns extend toward the open end of the proximal centriole and sharply bend around it [12]. Together, they create a vault wall around both the proximal and distal centrioles [12, 19]. This vault wall material is found embedded in the proximal centriole between the triplet microtubules [26, 51]. In contrast, the distal centriole appears unconnected to the capitulum or striated columns.

Caudally, the striated columns are connected to the outer dense fibers (Fig. 1b). Although the nine longitudinal striated columns are extended caudally and fused with the outer dense fibers [12, 52], the striated columns and outer dense fibers have a distinct origin and structural appearance. They form independently in the spermatids and fuse at the outer dense fibers' rostral end during spermatogenesis [52]. The striated columns are composed of alternating pale and dark bands, resulting from differences in electron density detected by transmission electron microscopy. In contrast, the outer dense fibers have a homogeneous appearance. The outer dense fibers extend alongside the midpiece axoneme and connect to the proximal half of the principal piece axoneme via specialized attachment points [46], caudally tapering in thickness [53], and end in the distal half of the principal piece [26, 46].

### Intracytoplasmic sperm injection (ICSI) may overcome acephaly-based infertility

It is essential to note the several significant morphological, structural, and functional differences between human (and most mammals) spermatozoa and mouse spermatozoa [54]. First, the mouse spermatozoon neck is attached to the side of the nucleus, rather than its base. Second, the mouse spermatozoon neck still retains a capitulum and striated columns, whereas the centrioles are unrecognizable; however, some centriolar proteins remain [46, 54, 55]. Third, in contrast to human spermatozoa, the injection of solely the mouse spermatozoon head into the egg is sufficient to produce offspring [56]. This head-injection only is due to the paternal centrosome being dispensable for mouse embryo development [57], unlike humans, where ICSI with the spermatozoa head only is unable to yield a diploid embryo [58]. Finally, while not affecting neck structure, mouse spermatozoa exhibit tails (~ 120 µm) that are about twice as long as human spermatozoa tails (~ 50 µm), and mouse spermatozoa have a distinct sickle-shaped head compared to human spermatozoa’s acorn/paddle-shaped heads [53]. These differences are significant and must be considered when comparing findings from studies of mouse and human spermatozoa mutations and ICSI outcomes.

Patients with few spermatozoa that cannot swim efficiently to the egg are commonly treated with ICSI [59]. ICSI in humans typically involves the injection of whole spermatozoon, and rarely, a spermatozoon fragment that includes only the head. Higher rates of fertilization are observed with whole spermatozoon rather than just the spermatozoon head [60]. ICSI revolutionized male infertility by allowing the injection of a single spermatozoon into an egg [61]; however, there are still times when couples are not able to conceive even with ICSI treatment. Even with normal morphological spermatozoa, ICSI may fail, suggesting other potential problems with the spermatozoa. Therefore, before ICSI, further testing with a patient’s spermatozoa’s DNA integrity, PLCζ quality, and centriole functionality should be conducted.

Approximately half of couples who undergo ICSI are unable to achieve a live birth [62–67]. Interestingly, studies on abnormal spermatozoa head shape and ICSI outcomes have found that patients with mutated genes related to acrosome formation and acrosome attachment to the nucleus generally have good ICSI outcomes. In contrast, mutated genes related to proacrosomal vesicle transport, perinuclear theca, and manchette structure typically result in poor ICSI outcomes. Interestingly, some of the genes associated with poor ICSI outcomes can sometimes be rescued by ICSI plus artificial oocyte activation [68]. Here, we will investigate the correlation between ICSI outcomes and mutations in neck components, as well as the location of the acephalic spermatozoon breakpoint.

Mutations of spermatozoa neck components are associated with two types of morphological spermatozoon defects: acephalic spermatozoa and abnormal tail morphology, such as multiple morphological abnormalities of sperm flagella (MMAF). There is no definite clinical definition of acephalic spermatozoa, but they are referenced as acephalic, or pinhead, spermatozoa in the World Health Organization laboratory manual [59]. The condition where the spermatozoa head is detached from the tail has also been described as headless, decapitated, decaudated, and pinhead spermatozoa (Fig. 1c). In a research setting, Acephalic Spermatozoa Syndrome (ASS) is the most widely accepted definition of acephalic, or decapitated, spermatozoa [22], and acephalic spermatozoa fall under the teratozoospermia classification [69]. It is estimated that the prevalence of acephalic spermatozoa is less than 0.1% [70, 71], but this is only a rough estimate, as there has been no large-scale study done on the rate of acephalic spermatozoa.

Multiple morphological abnormalities of sperm flagella are associated with many proteins in the tail and are less frequently associated with proteins in the neck; therefore, they are not the focus of our study [72, 73]. The neck proteins that cause MMAF or abnormal head morphology (ABH) when mutated are CETN1 [74, 75], POC1B [76], CEP128 [77], CEP78 [78], CEP135 [79, 80], SPAG4 [81, 82], and SDCCAG8 [83]. Heterologous ICSI studies in CETN1-mutated spermatozoa had poor embryonic spindle formation. ICSI studies with CEP128 and CEP78-mutated spermatozoa had unsuccessful pregnancies in humans and mice. ICSI studies with CEP135-mutated spermatozoa had unsuccessful pregnancies in humans. POC1B, SPAG4, and SDCCAG8 mutated spermatozoa lack ICSI studies. Overall, spermatozoa neck-associated MMAF appears to have poor ICSI outcomes; however, further research is needed on this subject.

### Acephalic spermatozoa are categorized based on precise neck breakpoints

**Fig. 2:**
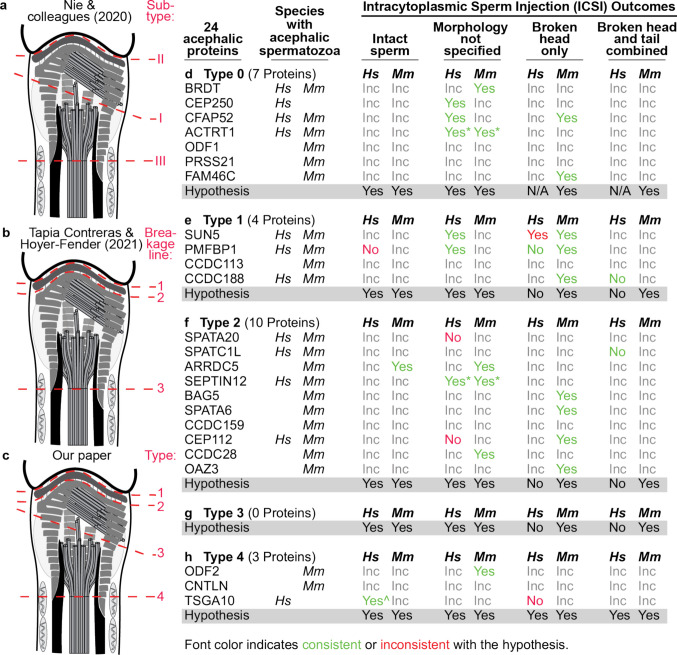
ICSI outcomes may be estimated by neck breakpoint. (**a-c**) Human spermatozoon neck proteins can be categorized based on the acephalic anatomical breakpoint and protein location. Breakpoint categorization by Nie and colleagues (2021) (**a**), Tapia Contreras & Hoyer-Fender (2021) (**b**), and our paper (**c**)**.** (**d-h**) Categorization of the 24 acephalic proteins to Type 0 (**d**), Type 1 (**e**), Type 2 (**f**), Type 3 (**g**), Type 4 (**h**); the species they were studied in (Hs, human; Mm, mouse); and ICSI outcomes. The categories' ICSI outcomes are indicated by a “Yes” label, indicating that ICSI successfully yielded at least one successful pregnancy in the study or blastocyst formation (in mice). A “No” label indicates that ICSI was unsuccessful. “Inc” (incomplete) means that the experiment has not been performed. “Morphology not specified” demonstrates that the literature did not specify whether the spermatozoa used for ICSI had flagella attached during ICSI. In this case, we assume the experimenters used intact spermatozoa for ICSI. The asterisk on ACTRT1 and SEPTIN12 indicates that ICSI was only successful after artificial activation of the oocyte. The caret on TSGA10 is due to the authors specifying they used intact spermatozoa when available, but may have also used combined head and tail. The Hypothesis row indicated our expected outcome based solely on the breakpoint. Green font “yes “ or “no” indicates that the experiment supports the hypothesis. Red font “yes “ or “no” indicates that the experiment is inconsistent with the hypothesis

Acephalic spermatozoa occur when the connecting piece fails to connect the head to the tail during spermiogenesis, resulting in a break somewhere within the neck (Fig. 2a-c) [22, 69, 84]. A literature search conducted using PubMed and Google Scholar databases until September 2025 revealed that 24 proteins (Table 1) were implicated in the formation of acephalic spermatozoa in humans and mice (Fig. 2d-h). Ten were studied in both humans and mice with a similar acephalic phenotype. Two were only studied in humans (CEP250 and TSGA10) and will benefit from confirmation in a model system. Twelve proteins were studied only in mice, and it will be clinically beneficial to confirm if similar phenotypes appear in humans. Of all 24 proteins studied, the types were consistent in both humans and mice and are therefore described together. Additionally, the proteins studied in mice and rats came from targeted knockout experiments. This suggests that studies in mice acephalic spermatozoa breakpoint types are informative for human diagnostic value.

**Table 1:** Abbreviations for acephalic proteins

Abbreviation	Full Protein Name	Aliases
BRDT	bromodomain testis associated	CT9
CEP250	centrosomal protein 250	CNAP1
CFAP52	cilia and flagella associated protein 52	WDR16
ACTRT1	actin related protein T1	AIP1
ODF1	outer dense fiber of sperm tails 1	RT7; ODF27; HSBP10
PRSS21	serine protease 21	Testisin
FAM46C	family with sequence similarity 46 member C	TENT5C
SUN5	sad1 and UNC84 domain containing 5	SPAG4L
PMFBP1	polyamine modulated factor 1 binding protein 1	STAP
CCDC113	coiled-coil domain containing 113	CFAP263
CCDC188	coiled-coil domain containing 188	
SPATA20	spermatogenesis associated 20	SSP411
SPATC1L	spermatogenesis and centriole associated 1	C21orf56
ARRDC5	arrestin domain containing 5	
SEPTIN12	septin 12	
BAG5	bag cochaperone 5	
SPATA6	spermatogenesis associated 6	SRF1
CCDC159	coiled-coiled domain containing 159	
CEP112	centrosomal protein 112	CCDC46
CCDC28	coiled-coil domain containing 28	C6orf80; LTAP2A; CCRL1AP
OAZ3	ornithine decarboxylase antizyme 3	AZ3; OAZ-T
ODF2	outer dense fiber of sperm tails 2	Cenexin;ODF84
CNTLN	centlein	C9orf39; C9orf101
TSGA10	testis specific 10	CEP4L; CT79

Five proteins (CNTROB, DNAH6, HOOK1, IFT88, and ARL3) (Table 2) that were previously suggested to cause acephalic spermatozoa were excluded from this study due to a lack of supporting evidence or a low spermatozoa acephalic rate (less than 50% acephalic spermatozoa). We excluded the CNTROB protein because the acephalic phenotype is questionable, as it was observed in a naturally occurring hypodactylous mutation in a rat that produced a truncated CNTROB protein [85]. However, after overexpression of the full-length CNTROB protein, the hypodactylous rat still produced acephalic spermatozoa, which the authors attributed to a mixture of truncated and full-length CNTROB protein [85, 86]. We also excluded the acephalic proteins that, when mutated, cause a weak acephalic phenotype, such as DNAH6 [87] and HOOK1 [88, 89]. Additionally, although Intraflagellar Transport 88 Protein (IFT88) has been characterized as an acephalic protein before [90], the original papers describing IFT88 mutations in mice primarily represent the mutation as affecting axoneme development, resulting in short and stumpy tails [91]. Therefore, we also omitted IFT88 as an acephalic protein in this review. Lastly, researchers used in vivo siRNA to reduce ARL3 expression in mice and found that spermatozoa from these experimental mice exhibited morphological defects, including a coiled tail, abnormal head shape, and some acephalic spermatozoa [92]. Therefore, we excluded ARL3 because further studies are needed to define its role.

**Table 2:** Abbreviations for excluded acephalic proteins

Abbreviation	Full Protein Name	Aliases
CNTROB	centrobin	LIP8
DNAH6	dynein axonemal heavy chain 6	DNHL1
HOOK1	hook microtubule tethering protein 1	HK1
IFT88	intraflagellar transport 88	Polaris Homolog
ARL3	ADP ribosylation factor like GTPase 3	ARFL3

Acephalic spermatozoa were categorized into distinct groups depending on the precise breakpoint in the spermatozoon neck by two papers around the same time [12, 23]. Both had three categories, but they differ in the naming order and where the breakpoint occurs. In the first paper, *Nie and colleagues (2020)* (Fig. 2a) defined a break between the proximal and distal centrioles as subtype I, a break between the nuclear envelope and the basal plate as subtype II, and the separation of the distal centriole from the midpiece as subtype III. They suggested that categorizing acephalic spermatozoa can aid in the diagnosis of ICSI outcomes [23]. Cazin and colleagues [93] also published a paper describing the same break types about a year later.

In the second paper, *Tapia Contreras and Hoyer-Fender (2021)* (Fig. 2b) defined a break between the nuclear envelope and the basal plate as breakage line 1, a break between the basal plate and the proximal centriole as breakage line 2, and, like the first paper, a break below the distal centriole as breakage line 3 [12].

While both research groups make strides in categorizing acephalic breaks, both systems fail to account for one of the four possible breaks; Nie and colleagues (2020) do not account for a break between the basal plate and proximal centriole, and Tapia Contreras and Hoyer-Fender (2021) do not account for a possible break between the proximal and distal centrioles. Additionally, Nie and colleagues (2020) propose a naming mechanism that is not ordered rostral to caudal. Therefore, we have surveyed the literature on human and mouse acephalic spermatozoa studies (Fig. 2) and propose systematically organizing five types (types 0, 1, 2, 3, and 4) of acephalic spermatozoa based on the breakpoint relative to spermatozoon neck substructures in a rostral-to-caudal order (Fig. 2c). For this categorization, we utilize published electron microscopy studies of mutant spermatozoa and spermatids.

*Type 0* refers to any acephalic phenotype that is caused without a consistent or known breakpoint. This type has seven proteins: BRDT [94], CEP250 [95], CFAP52 [96], ACTRT1 [70, 97], ODF1 [98–100], PRSS21 [101], and FAM46C [102, 103] (Fig. 2d). BRDT, CFAP52, and ACTRT1 were studied in humans and mice; CEP250 was studied only in humans; and ODF1, PRSS21, and FAM46C were studied only in mice. Interestingly, while fresh ejaculate from ODF1-deficient men did not have acephalic spermatozoa, after a freeze-thawing cycle, most human spermatozoa were acephalic [98]. This acephaly from ODF1-mutated spermatozoa could be due to a poor cryopreservation technique.

While Nie and colleagues (2020) classify BRDT as a subtype III (analogous to our type 4 protein) [23], we classify BRDT as a type 0 protein, as both papers describing BRDT mutations [94, 104] do not describe the break type ultrastructurally. Li and colleagues (2017) describe the mitochondrial sheath as missing from acephalic spermatozoa, but do not specify the location of the break [94]. Nie and colleagues (2020) describe that the acephalic spermatozoa broke below the distal centriole.

*Type 1* is the separation between the nucleus and the basal plate. In this case, the head, with its nucleus and nuclear membrane, is separated from the tail, which contains the basal plate and all other neck structures (Fig. 2e). Type 1 comprises four proteins: SUN5 [105], PMFBP1 [106], CCDC188 [107, 108], and CCDC113 [109]. SUN 5, PMFBP1, and CCDC188 were studied in both humans and mice, while CCDC113 was studied only in mice. This is analogous to Nie and colleagues’ subtype II and Tapia Contreras & Hoyer-Fender’s breakage line 1.

*Type 2* is a separation between the basal plate and the proximal centriole. In this case, the head, with its nucleus and basal plate and possibly part of the capitulum, is separated from the tail, which contains most of the connecting piece (capitulum and striated columns) and centrioles (Fig. 2f). Type 2 has ten proteins: CEP112 [110, 111], SPATA20 [112], SPATC1L [113], ARRDC5 [114], BAG5 [37], CCDC159 [115], SEPTIN12 [116], SPATA6 [117], CCDC28 [118], and OAZ3 [119]. Four proteins, SPATA20, SPATC1L, SEPTIN12, and CEP112, were studied in both humans and mice, while zero proteins were studied in humans alone. Six proteins, ARRDC5, BAG5, SPATA6, CCDC159, CCDC28, and OAZ3, were studied only in mice. CNTROB was only studied in rats and would fit this category if the study were conclusive regarding the mutated protein [85, 86]. Nie and colleagues do not have this as a separate category; however, this category is analogous to Tapia Contreras & Hoyer-Fender’s breakage line 2.

*Type 3* is a separation between the proximal and distal centrioles. In this case, the head, with its nucleus, basal plate, capitulum, and possibly part of the striated columns, is separated from the distal centriole, the axoneme, outer dense fibers, and the remainder of the tail (Fig. 2g). Only one case of Type 3 was described in a young man who underwent a testicular biopsy, revealing that the absence of striated columns led to a break between the two centrioles during spermiogenesis, resulting in a fully formed head and tail [120]. However, Type 3 has zero identified proteins. FAM46C may be a candidate protein for Type 3, but we still classify it as a Type 0 due to inconclusive evidence. The authors demonstrated that FAM46C-mutated mice spermatozoa exhibited incomplete striated column formation, similar to other Type 3 cases, and their transmission electron microscopy images revealed acephalic spermatozoa with the proximal centriole aligned with the head and the distal centriole still attached to the axoneme [102]. Therefore, we propose that if a mutation affects the striated columns’ formation, this will serve as a possible mechanism for a Type 3 acephalic break.

The lack of identified proteins is remarkable, as in somatic cells, a set of centrosomal/centriolar linker proteins, including C-Nap1, Cep135, Cep68, Cep215, LRRC45, Rootletin, CNTLN, CCDC102B, and LGALS3BP, are implicated in forming the linker and maintaining the connection between the two centrioles of the centrosome [121, 122]. Interestingly, Nie and colleagues also identified their subtype I as a breakpoint between the proximal and distal centrioles, but Tapia Contreras and Hoyer-Fender did not.

*Type 4* is a separation below the distal centriole, somewhere along the proximal portion of the axoneme. In this case, the head, with its nucleus, basal plate, capitulum, striated columns, the two centrioles, and possibly a part of the outer dense fibers and axoneme, is separated from the remainder of the tail (Fig. 2h). Subtype 4 comprises three proteins: ODF2 [123], CNTLN [124], and TSGA10 [125]. One of these proteins, TSGA10, was tested in humans. CNTLN and ODF2 exhibited an acephalic phenotype in mice but have not yet been studied in humans. This is analogous to Nie and colleagues’ subtype III and Tapia Contreras & Hoyer-Fender’s breakage line 3.

### Acephalic spermatozoa protein is localized within and outside the spermatozoa neck

To determine the connection between acephalic type and protein localization, we searched the literature for descriptions of the acephalic protein localization in spermatozoa (Table 3). As expected from a structural role in connecting the head to the tail, the majority of acephalic proteins (18 of the 24) were localized to the neck. There are nine proteins that are primarily detected in the neck. This includes type 0 protein, CEP250 [95]; type 1 proteins, SUN5 [105], PMFBP1 [106, 126], and CCDC188 [108]; type 2 protein SPATA20 [112], BAG5 37, SPATA6 [117], CEP112 [110], and CCDC28 [118].

**Table 3:** Acephalic spermatozoa proteins are mainly localized to the spermatozoon neck

Protein	Protein Type	Category	Spermatozoa Localization		Spermatid Localization		Spermatozoa Localization*
			*Hs*	*Mm*	*Hs*	*Mm*	*Other Species*
BRDT	Type 0	Head	Incomplete	Incomplete	Incomplete	Head [135, 136]	Incomplete
CEP250	Type 0	Neck	Neck [95]	Incomplete	Incomplete	Incomplete	Incomplete
CFAP52	Type 0	Neck & Other	Neck/Tail [96]	Incomplete	Incomplete	Incomplete	Incomplete
ACTRT1	Type 0	Neck & Other	Nu [97]; Neck/MP [70]	Incomplete	Incomplete	Acr [138]	Incomplete
ODF1	Type 0	Neck & Other	Incomplete	Tail [127]	Incomplete	Man [127]	SCs/Cap/ODFs (*Rn* ES)^%^ [50]
PRSS21	Type 0	Neck & Other	Incomplete	Neck/CD [33]	Incomplete	Incomplete	Neck/Tail (*Ec*) [128]
FAM46C	Type 0	N/A	Incomplete	Incomplete	Incomplete	Incomplete	Incomplete
SUN5	Type 1	Neck	Neck [90, 96, 105]; NuE^%^ [35]	IF [105]; Neck [106, 108]	Incomplete	Neck [105]	Neck (*Bt, Oa, Rn*) [105]
PMFBP1	Type 1	Neck	Neck [106, 126, 132]	Neck [106, 108, 126]	Incomplete	IF [106]	Incomplete
CCDC113	Type 1	Neck & Other	Neck/Tail [109]	Neck/Tail [109]	Incomplete	Neck/Tail/Man [109]	Incomplete
CCDC188	Type 1	Neck	Neck [108, 139]	Incomplete	Incomplete	Incomplete	Incomplete
SPATA20	Type 2	Neck	Neck [112]	Incomplete	Incomplete	Cyt/Neck [112]	Incomplete
SPATC1L	Type 2	Undetected	Undetected [137]	Undetected [113]	Neck [137]	Neck [113]	Incomplete
ARRDC5	Type 2	N/A	Incomplete	Incomplete	Incomplete	Incomplete	Incomplete
SEPTIN12	Type 2	Neck & Other	Neck/Ann [116]	Neck [116]	Incomplete	Neck [116]	Incomplete
BAG5	Type 2	Neck	BP [37]	BP [37]	Incomplete	BP/Man^%^ [37]	BP (*Bt, Cp, Rn, Ss*) [37]
SPATA6	Type 2	Neck	Neck [96, 106, 117]	Neck [106]; SCs/Cap^%^ [117]	Incomplete	Cyt/Neck [112]; Man [117]	SCs/Cap^%^ (*Oc*); Neck (*Mf, Om, Rn*) [117]
CCDC159	Type 2	Undetected	Incomplete	Undetected [115]	Incomplete	Neck [115]	Incomplete
CEP112	Type 2	Neck	DC^%^ [110]	Neck [110]	Cyt [110]	Cyt/Neck [110]	DC (*Bt, Ss*) [110]
CCDC28	Type 2	Neck	Incomplete	Incomplete	Incomplete	Man/Neck [118]	Incomplete
OAZ3	Type 2	Neck & Other	Incomplete	Tail [129]	Incomplete	Tail [129]	SCs/Cap/ODFs/FS^%^ (*Rn*) [129]
ODF2	Type 4	Neck & Other	Neck/MP/PP [130]	Incomplete	Incomplete	Incomplete	SCs/Cap/ODFs^%^ (*Rn* ES) [50, 131]
CNTLN	Type 4	Undetected	Incomplete	Undetected [124]	Incomplete	PC/DC [124]	Incomplete
TSGA10	Type 4	Neck	Neck [125, 132]; PC/DC [132]; MP [133]	Neck/MP [134]	Incomplete	Incomplete	Neck/MP (*Bt*) [134]

Annulus (Ann), acrosome (Acr), basal plate (BP), cytoplasm (Cyt), distal centriole (DC), nuclear envelope (NuE), fibrous sheath (FS), midpiece (MP), principal piece (PP), proximal centriole (PC), implantation fossa (IF), and the spermatid-specific manchette (Man). 'Undetected' indicates localization was tested but not found. 'Incomplete' indicates localization was not tested. In the *Other Species* Spermatozoa Localization Column, an asterisk denotes that there were two proteins (ODF1 and ODF2) that were studied in rat elongating spermatids (ES). Protein localization, characterized by ultrastructural techniques is denoted by a percent sign (%). *Bos taurus* (Bt); *Cavia porcellus* (*Cp*); *Equus caballus* (Ec); *Macaca fascicularis* (Mf); *Oryctolagus cuniculus* (Oc); *Oncorhynchus mykiss* (Om); *Ovis aries* (Oa); *Rattus norvegicus* (*Rn*); and *Sus scrofa* (*Ss*)

Nine proteins were identified in the neck and other regions of the spermatozoon. This includes type 0 proteins, CFAP52 [96]; ACTRT1, which is localized to the lower half of the spermatozoon nucleus [97] and with a PCM protein in the neck and midpiece [70]; ODF1 [50, 127] (in rat and mice); and PRSS21 found in the mice spermatozoon cytoplasmic droplet [33] and in the stallion spermatozoon neck and tail [128]. Type 1 protein CCDC113 [109] is also found in the tail. Type 2 proteins SEPTIN12 [116] and OAZ3 [129] are also found in the tail. OAZ3 was found to localize to the rat capitulum, striated columns, outer dense fibers, and fibrous sheath [129]. Lastly, two type 4 proteins, ODF2 (in rats and humans) [50, 130, 131] and TSGA10 [125, 132–134] are also found in the tail.

Interestingly, a type 0 acephalic protein, BRDT, was mapped primarily to the head. BRDT is a testis-specific protein that regulates gene expression by influencing chromatin structure and transcription [104]. It is localized to the nucleus in mouse spermatids [135, 136]. It may result in the expression of genes that control the head-to-tail connection.

Three acephalic proteins were studied but remained undetected in the spermatozoa, presumably because their essential function is during spermiogenesis. This includes type 2 proteins, SPATC1L [113, 137] and CCDC159 [115], and type 4 protein, CNTLN [124]. Interestingly, all are detected in the spermatid neck during spermiogenesis, suggesting a potential explanation for the acephalic phenotype.

Two protein localizations were not studied in the spermatozoa: FAM46C and ARRDC5. While FAM46C (type 0) [102] and ARRDC5 (type 2) [114] were studied, neither protein has undergone any immunofluorescence or localization studies in spermatozoa.

The localization of only seven acephalic proteins was studied at the subcellular resolution in the spermatozoa neck, and they include one type 0 protein, one type 1 protein, four type 2, and one type 4 acephalic protein. All used immunogold electron microscopy, except for SUN5, which used the more precise Cryo-EM technology, which is promising in replacing immuno-electron microscopy.

ODF1 (type 0) and ODF2 (type 4) were found to be components of the rat spermatid basal plate, striated columns, outer dense fibers, and mitochondrial sheath [50]. SUN5, a type 1 protein, was recently found to form a two-dimensional lattice in the nuclear envelope, just above the basal plate, using in situ cryo-electron tomography [35, 36]. Four type 2 proteins were analyzed at the subcellular resolution. BAG5 was found in the mouse spermatid neck’s basal plate [37]. SPATA6 was found in the mouse epididymal and rabbit ejaculated spermatozoa capitulum and striated columns [117]. CEP112 was found in the human spermatozoa distal centriole and the axonemal microtubules right below the distal centriole [110]. OAZ3 was found in the rat capitulum, striated columns, and outer dense fibers [129].

### Acephalic spermatozoon types may be used to predict the outcome of ICSI

We asked about the relevance of the acephaly categories and intracytoplasmic sperm injection (ICSI) outcomes. It is essential to note that the acephalic phenotype is typically incomplete, and some intact spermatozoa in which the head is connected to the tail can still be present and used for ICSI. Alternatively, a head-only or a combination of a separated head and tail can be used for ICSI. Therefore, we expect that intact spermatozoa of all acephalic types or type 4 spermatozoa heads can produce offspring with ICSI, unless the mutation also negatively affects the DNA, PLCζ, or centriole functionality.

Some of the manuscripts we reviewed do not explicitly indicate whether they injected intact spermatozoa or their parts; in these cases, we will assume that they injected intact sperm. Overall, we found that ICSI information is available for 19 out of 24 proteins identified. They comprise seven in humans and mice, including CFAP52 [96], ACTRT1 [70, 97], SUN5 [90, 105], PMFBP1 [140], CCDC188 [108, 139], SEPTIN12 [141, 142], and CEP112 [110].

They also include four proteins ICSI studied in humans only, namely CEP250 [95], SPATA20 [112], SPATC1L [137], and TSGA10 [125]. A mutant CEP250 mouse model was generated, and it was found to exhibit non-obstructive azoospermia, displaying species-specific phenotypes of CEP250 [95], which explains why it was not previously studied with ICSI in mice.

Finally, they include eight proteins studied with ICSI in mice only: BRDT [104], FAM46C [102], ARRDC5 [114], BAG5 [37], SPATA6 [117], CCDC28 [118], OAZ3 [119], and ODF2 [123]. It will be essential to obtain ICSI information on ODF1, PRSS21, CCDC113, CCDC159, and CNTLN, the remaining five proteins that lack ICSI studies in either humans or mice. While BRDT acephalic spermatozoa were recognized in a male patient, the patient did not undergo any fertility studies with the mutated spermatozoa [94]. While acephalic spermatozoa breakpoint types are consistent across species, ICSI outcomes are different in humans and mice, because human spermatozoa require their centrioles for ICSI, whereas mouse spermatozoa do not. Therefore, ICSI outcomes are described separately, below.

#### ICSI with intact spermatozoa from acephalic spermatozoa syndrome patients can still produce offspring

It is expected that if acephalic spermatozoa have a specific defect that only breaks the head-tail connection and does not affect other essential spermatozoon functions (DNA, PLCζ, or centrioles), then ICSI with the whole intact human spermatozoon will produce offspring. Indeed, this is the case for five of the 11 proteins tested with ICSI. These five proteins include CEP250 [95], CFAP52 [96], ACTRT1 [97], SUN5 [105, 143, 144], and SEPTIN12 [141]. Interestingly, PMFBP1 yielded mixed results [126, 132, 140]. CCDC188 [139], SPATA20 [112], SPATC1L [137], CEP112 [110], and TSGA10 [125] were unsuccessful (Table 4).

**Table 4:** ICSI outcomes with human acephalic spermatozoa proteins

Protein	Zygote Formation	Successful Implantation	Successful Pregnancy	Live Birth
CEP250	**Yes**	Incomplete	Incomplete	**Yes**
CFAP52	**Yes**	Incomplete	Incomplete	**Yes**
ACTRT1	**Yes**	Incomplete	**Yes**	Incomplete
SUN5	**Yes** [143, 144]	Incomplete	**Yes** [105, 143, 144]	**Yes** [105, 143, 144]
PMFBP1	**Yes** [106, 132, 140]	Incomplete	**Yes** [106, 132] **No** [132, 140]	**Mixed** [106, 132]
CCDC188	**Yes**	Incomplete	**No**	Incomplete
SPATA20	**Yes**	**No**	**No**	Incomplete
SPATC1L	**Yes**	**No**	Incomplete	Incomplete
SEPTIN12	**Yes**	Incomplete	Incomplete	Incomplete
CEP112	**Yes**	**Yes**	**No**	Incomplete
TSGA10	**Yes** [125, 132]	**Yes** [125, 132]	**Mixed** [132] **No** [125]	**Yes** [132] **No** [125]

Incomplete indicates that the information was not explicitly provided in the paper. Yes indicates a successful result. No indicates an unsuccessful result. Mixed indicates multiple patients were tested, and some had “yes” and some had “no” results. If no references are given, they only have one source.

Mutants in three *type 0* proteins were able to produce offspring with ICSI (CEP250, CFAP52, and ACTRT1). Because the precise breakpoint is not defined, the relative position of the centrioles is unknown, and predicting the diagnosis in humans based on the breakpoint is not possible based on the criteria established in this review. Interestingly, ACTRT1-mutated spermatozoa in a male patient had decreased PLCζ expression, and ICSI was successful when paired with artificial oocyte activation [97].

In *type 1*, we expect that intact spermatozoa will produce offspring with ICSI. In contrast, using only the head, only the tail, or a combination of head and tail that are separated from each other, will fail to produce offspring with ICSI. However, the literature is inconsistent with this hypothesis. As expected, ICSI with intact SUN5-mutated spermatozoa produced offspring in two studies [143, 144]. Unexpectedly, in one of these studies, ICSI with SUN5-mutated spermatozoa heads only also resulted in the production of an offspring in two different families with SUN5 mutations, contradicting our initial hypothesis [105, 143, 144]. PMFBP1-mutated intact spermatozoa produced offspring in two studies, as expected [106, 132]. In another study, a PMFBP1 patient was unable to produce a pregnancy, but the patient’s spermatozoa had abnormal tail morphology in their entire spermatozoa population [140]. As expected from our hypothesis, ICSI with PMFBP1-mutated spermatozoa heads only failed to produce offspring [140]. Lastly, ICSI with CCDC188-mutated intact spermatozoa did not yield an offspring, contrary to our hypothesis, even with artificial oocyte activation [139]. Although the authors noted that they used a combined, separated head and tail, spermatozoon, when intact sperm could not be used. They did not specify which spermatozoon morphology was used for each ICSI cycle; therefore, we are categorizing CCDC188 ICSI as intact sperm.

In *type 2*, we expect that intact spermatozoa from the mutated individual would produce offspring with ICSI. Unexpectedly, ICSI with intact spermatozoa from the SPATA20 mutant failed to make an offspring in one study [112]. In this study, a nonsense mutation was identified, but the effect on spermatozoon DNA was not investigated. ICSI performed with SPATC1L-mutated spermatozoa did not result in a successful pregnancy, with the authors claiming a centriole defect [137], but they did not check for DNA or PLCζ function. Additionally, intact spermatozoa from haploinsufficient SEPTIN12 male patients did not produce offspring after ICSI. However, they were able to produce offspring after a combination of ICSI and artificial oocyte activation, and a likely cause may be due to abnormal PLCζ levels in these patients [141]; however, the authors did not test PLCζ expression in the patient. Therefore, SEPTIN12-affected patients may need to go through ICSI and PLCζ treatment to achieve a successful pregnancy. Lastly, CEP112-mutated patient spermatozoa did not yield a successful pregnancy, with the authors explaining that CEP112 may influence early embryo development. They noted the limited sample size, which precluded drawing any conclusions [110]. They suggested performing a centriole quality evaluation, such as Fluorescence-Based Ratiometric Analysis of Sperm Centrioles [145].

In* type 4*, we expect ICSI with intact spermatozoa or acephalic spermatozoa to produce offspring. Only TSGA10 has been studied in humans, in two different instances. While ICSI with intact TSGA10 spermatozoa produced healthy offspring in two couples [132]; unexpectedly, ICSI with TSGA10-mutated spermatozoa heads only failed to produce an offspring [125], contradicting our hypothesis. In the latter case, ICSI produced a human embryo that was unable to implant; unfortunately, there was limited discussion on any other essential factors, such as DNA or PLCζ function. The authors explained the failure as possibly due to abnormalities in the spermatozoon-derived centrosome.

It is important to note that there are a multitude of reasons why a couple is unable to achieve a successful pregnancy, even after using ICSI. There are both technical challenges and biological factors. Technical challenges include the handling of male and female gametes, the ICSI technique, and post-ICSI procedures [146]. Biological factors are due to the newly formed zygote facing multiple challenges to develop into a live birth, including blastocyst formation, successful implantation, and a successful pregnancy [146]. Additionally, several female factors may be involved that can hinder zygote formation, including age, genetic, and physical factors [147]. All of these challenges contribute to the complexity of clinical outcomes and should be reported, if available, during ICSI studies. While ICSI and in vitro fertilization (IVF) are often used to treat infertility, couples can also pursue alternative treatments such as round spermatid injection (ROSI) [148, 149] or elongating/elongated spermatid injection (ELSI) [150]. These methods can also be paired with testicular sperm extraction (TESE) if spermatozoa are unable to be found [151].

#### All types of acephalic spermatozoa can produce offspring with ICSI in mice

Mouse spermatozoa exhibit a distinct morphology and neck structure compared to those of non-murine mammalian species. Most significantly in mice, unlike non-murine mammalian spermatozoa, centrioles are dispensable post-fertilization, and injection of the nucleus into the oocyte is sufficient for obtaining mouse offspring [152]. In line with this established dogma, ten described mutant acephalic spermatozoa were capable of producing offspring via ICSI of the mouse spermatozoon head only. These proteins include CFAP52 [96], FAM46C [102], SUN5 [105], PMFBP1 [106], CCDC188 [108], SEPTIN12 [141], BAG5 [37], SPATA6 [117], CEP112 [110], and OAZ3 [119]. Also, five out of the five described mutant acephalic spermatozoa could produce offspring via ICSI when used with the whole spermatozoon (mutations in BRDT, ACTRT1, ARRDC5, CCDC28, and ODF2).

While FAM46C ICSI with spermatozoa heads only was able to produce an offspring, it was a very low yield, at only 1 in 233 embryos producing a live mouse. This low efficiency is possibly due to apoptosis-induced DNA damage, as confirmed by the acridine orange test, rather than improper chromatin condensation [102].

SEPTIN12 is likely involved in forming the nuclear envelope, as suggested by its localization during human spermiogenesis and its mutant phenotype [142]. The SEPTIN12 complex is required for the establishment of a functional spermatozoon head–tail junction [116]. Interestingly, ICSI failed to produce offspring in spermatozoa from homozygous mice with SEPTIN12 mutations, but was recoverable by a combined ICSI and artificial oocyte activation method, like humans [141]. This ICSI failure may have been due to the loss of PLCζ expression in SEPTIN12-mutated mice spermatozoa [141]. Similarly, ICSI of heterozygous SEPTIN12 mouse spermatozoa failed due to significant DNA damage, as assessed by acridine orange and toluidine blue for DNA integrity and aniline blue for DNA condensation [142]. Still, another set of heterozygous SEPTIN12 mice, analogous to the heterozygous male patient, were found to be fertile [141].

Additionally, ACTRT1-mutated spermatozoa can be rescued with ICSI and artificial oocyte activation, following the observation of decreased levels of PLCζ expression [97]. ACTRT1 is a protein that localizes to the acrosome in mouse spermatozoa and connects the acrosome to the nucleus; knockout mouse models show decreased PLCζ expression [138]. The above findings suggest that in mice, ICSI can overcome all acephalic spermatozoon types, as long as they do not have damage to DNA and/or PLCζ, with centrioles not being required for mouse ICSI.

### Other neck proteins are candidate acephalic proteins

**Fig. 3:**
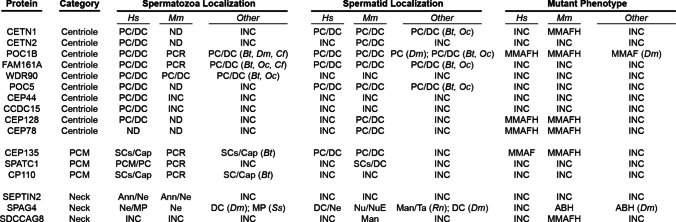
Proteins localize to the spermatozoon neck and can potentially cause acephalic spermatozoa. Structure names are shown in Fig. 1. The manchette (Man), cytoplasm (Cy), and nuclear envelope (NuE). ND indicates not detected, and INC (incomplete) indicates the research has not been done yet. PCR refers to the proximal centriole remnant. We used the term 'tail' as a preferred synonym for the flagellum. *Bos taurus* (*Bt*); *Drosophila melanogaster* (*Dm*); *Canis familiaris* (*Cf*); *Oryctolagus cuniculus* (*Oc*); *Rattus norvegicus* (*Rn*); and *Sus scrofa* (*Ss*). Mutant phenotypes include MMAF (multiple morphological abnormalities of sperm flagella), MMAFH (multiple morphological abnormalities of sperm flagella and the head), and ABH (abnormal head morphology)

Other proteins localize to the spermatozoon neck and can potentially cause acephalic spermatozoa. Here we identified 16 such proteins (Fig. 3) (Table 5). Seven of these proteins have been found to cause morphological defects such as multiple morphological abnormalities of sperm flagella (MMAF), multiple morphological abnormalities of sperm flagella and the head (MMAFH), or abnormal head morphology (ABH) with little or no acephaly (CETN1, POC1B, CEP128, CEP78, CEP135, SPAG4, and SDCCAG8).

**Table 5:** Abbreviations for potential acephalic proteins

Abbreviation	Full Protein Name	Aliases
CETN1	centrin 1	
CETN2	centrin 2	
POC1B	POC1 centriolar protein B	WDR51B
FAM161A	FAM161 centrosomal protein A	
WDR90	WD repeat domain 90	C16orf15
POC5	POC5 centriolar protein	
CEP44	centrosomal protein 44	KIAA1712
CCDC15	coiled-coil domain containing 15	
CEP128	centrosomal protein 128	C14orf145
CEP78	centrosomal protein 78	C9orf81
CEP135	centrosomal protein 135	
SPATC1	spermatogenesis and centriole associated 1	SPATA15; SPRN
CP110	centriolar coiled-coil protein 110	KIAA0419
SEPTIN2	septin 2	
SPAG4	sperm associated antigen 4	SUN4
SDCCAG8	serologically defined colon cancer autoantigen protein 8	NPHP10

This includes ten proteins (CETN1, CETN2, POC1B, FAM161A, WDR90, POC5, CEP44, CCDC15, CEP128, and CEP78) that localize primarily at the centrioles (proximal centriole, distal centriole, or both), three proteins (CEP135, SPATC1, and CP110) that localize to the pericentriolar material (PCM, capitulum, or striated columns), and three proteins (SEPTIN2, SPAG4 and SDCCAG8) with a general localization in the neck region.

#### Ten spermatozoon proteins localize only to the centrioles

Of the ten proteins localized to the centriole, nine localized to both centrioles in the human spermatozoa, and one localized to both centrioles in the mouse spermatid. CETN1 [21] and CETN2 were localized in human and bovine spermatozoa to the proximal and distal centrioles [44]. Light and electron microscopy analyses found that CETN1 is essential for normal mouse spermatids’ centriole rearrangement and basal-body-nucleus connection [74]. CETN1 is undetected in mature mouse spermatozoa despite its earlier localization [74], nor is CETN2 present in mature mouse spermatozoa despite being found in caput epididymal spermatozoa [153].

CETN1 mutations cause abnormal head morphology in mice [74]. The authors did not perform any ICSI treatments. Interestingly, a group performed heterologous ICSI (bovine oocyte with human spermatozoa) and found that the patient’s spermatozoa had reduced levels of CETN1 and failed to produce a spindle in 90% of zygotes in CETN1-depleted spermatozoa populations [75].

POC1B was localized in human and bovine spermatozoa to the proximal centriole and distal centriole [44], the mouse proximal centriole remnant, and dog centrioles [54]. Drosophila POC1B ortholog has been found to localize to the proximal centriole-like structure in Drosophila spermatids [154, 155]. POC1B mutations have been linked to MMAF and abnormal head shapes in humans and mice [76] and their Drosophila ortholog for spermatozoon centriole formation and function [155]. ICSI studies have not been done with POC1B-mutated spermatozoa.

FAM161A was localized in human, bovine, and rabbit spermatozoa to the proximal and distal centrioles [16], as well as the mouse proximal centriole remnant and dog centrioles [54]. WDR90 was localized in human, bovine, and rabbit spermatozoa to the proximal and distal centrioles [16]. POC5 was localized in human and bovine spermatozoa to the proximal and distal centrioles [44]. CEP44 and CCDC15 were localized in human spermatozoa proximal and distal centrioles [156].

Additionally, CEP128 was found to be localized to the two centrioles in human spermatozoa, and mutant CEP128 in human and mice spermatozoa showed abnormal neck and tail development, but rarely did it show acephalic spermatozoa [77]. The authors performed ICSI using the patient’s spermatozoa without success, and with mice knock-in and knock-out models, also with poor ICSI outcomes. No functional testing on DNA, PLCζ, or centrioles was conducted, but they suggested that in mice, CEP128 mutations also had associated decreased gene expression in other proteins essential for embryonic development.

Interestingly, a CEP78 human patient and a knockout mouse model showed abnormal neck and tail development with staining at the centrioles in developing spermatids, but disappeared in mature spermatozoa [78], much like SPATC1L, CCDC159, and CNTLN. ICSI was performed with an unsuccessful pregnancy in humans, where the authors explained it as a centriole defect that hindered zygote development, as the sperm DNA fragmentation index was normal at 17%. Knockout mice were also unable to overcome ICSI, with the authors explaining that CEP78 plays another crucial role in embryonic development, as evidenced by the absence of DNA fragmentation or micronuclei, which was tested by acridine orange staining [78]. The study did not examine PLCζ function. Additionally, another study found a CEP78 mutation caused MMAF, abnormal head morphology, and, interestingly, found triplet microtubules in the axoneme, indicating centriole elongation, in both human and mouse spermatozoa [157]. The authors did not perform any ICSI experiments.

#### Three proteins localize to the pericentriolar material of the spermatozoon

Three proteins are localized to the spermatozoon pericentriolar material. CEP135 was localized to the striated columns and capitulum near the proximal and distal centriole in human and bovine spermatozoa [158], the proximal centriole remnant in mice [55], and in mouse spermatid centrioles [79]. Yet another study did not detect CEP135 in mature spermatozoa [79]. Mutating CEP135 causes MMAF in humans and ICSI experiments did not yield a successful pregnancy [80] and MMAFH in mice [79]. No functional testing on DNA, PLCζ, or centrioles was conducted, but the lack of ICSI success in humans was ascribed to centriole defects. SPATC1 was localized around the human spermatozoa proximal centriole, at the periphery of the remnant distal centriole in mouse spermatozoa, and at mouse spermatid striated columns [159], suggesting it localizes to the pericentriolar material of the sperm. CP110 was localized to the striated columns and capitulum in human and bovine spermatozoa [158] and in mouse spermatozoa near the proximal centriole remnant [55].

#### Three proteins localize generally in the spermatozoon neck

Three proteins only have a general localization in the neck. SEPTIN2 is localized to the neck and annulus of human [160] and mice spermatozoa [161]. Additionally, SPAG4 is localized diffusely throughout the neck in human spermatids and spermatozoa [162] as well as the manchette and the tail in rat spermatids [163]. SPAG4 mutant mice have abnormal spermatozoa heads [81, 82]. SPAG4 colocalizes with centriolar marker gamma tubulin in Drosophila spermatids, and mutant Drosophila have abnormal spermatozoon head morphology and incorrect nucleus-centriole connections [164]. Additionally, SPAG4 localizes to the midpiece of human and boar spermatozoa, as well as to the neck in mouse spermatozoa [35]. ICSI studies have not been done with SPAG4-mutated spermatozoa.

Interestingly, a centriolar satellite protein, SDCCAG8, was found to have transient localization to the centrioles in round spermatids and to the manchette in spermatids. Mutations in this protein cause MMAFH in mice spermatozoa [83]. ICSI studies have not been done with SDCCAG8-mutated spermatozoa.

The effects of mutation in many of the above-mentioned neck proteins have not been studied, or they are associated with other phenotypes characterized by low levels of acephaly. Due to their localization in the neck, these proteins are potential candidates to cause acephaly in spermatozoa and may benefit from additional clinical testing in the future.

## Conclusion

We have investigated the relationship between mutations in the spermatozoon neck proteins and ICSI outcomes. We found that spermatozoon neck proteins with abnormal tail morphology, such as MMAF, are associated with lower ICSI success rates. Acephalic phenotype ICSI outcomes are more variable.

We provided suggestions for classifying acephalic spermatozoa based on the neck breakpoints, describing 24 proteins in this review. The acephalic spermatozoon types can be diagnosed via electron microscopy; however, this information currently offers limited aid to the ICSI operator. For patients with acephalic spermatozoa of types 0, 1, 2, 3, and 4 that contain some intact spermatozoa or those with type 4 that have only spermatozoa heads, ICSI may be a feasible treatment option. This is contingent upon high DNA integrity, as evaluated through DNA fragmentation [7] and fluorescence in situ hybridization [165] testing, standard PLCζ localization/function as determined by diagnostics, such as antibody staining [166], and normal centriolar function via Fluorescence-Based Ratiometric Analysis of Sperm Centrioles (FRAC) testing [145, 167–169]. These assays would be diagnostic tests performed before treatment.

Furthermore, embryologists and ICSI operators may be able to employ future techniques that aid them in selecting quality spermatozoa during ICSI treatment, such as Artificial Intelligence-based spermatozoon morphology and motility assays [170], DNA quality assays based on spermatozoon head density [171], and centriole quality assays based on spermatozoon head kinking [16].

Overall, spermatozoa neck-based ICSI outcomes are complex. It involves several additional phenotypic parameters: spermatozoon DNA integrity, spermatozoon PLCζ functionality, and spermatozoon centriole quality. Therefore, we recommend that all patients diagnosed with acephalic spermatozoa have their spermatozoa examined using transmission electron microscopy to identify the type of breakpoints. Additionally, their DNA, PLCζ, and centriole quality should be tested.

## Data Availability

Data sharing is not applicable as no new datasets were generated.
